# Cardiology on the cutting edge: updates from the European Society of Cardiology (ESC) Congress 2020

**DOI:** 10.1186/s12872-020-01734-4

**Published:** 2020-10-19

**Authors:** Enrique Gallego-Colon, Aldo Bonaventura, Alessandra Vecchié, Antonio Cannatà, Ciarán Martin Fitzpatrick

**Affiliations:** 1 Cardiology Department, Barzilai University Medical Center, Hahistadrout St 2, 30604 Ashkelon, Israel; 2 Pauley Heart Center, Division of Cardiology, Department of Internal Medicine, Virginia Commonwealth University, 1200 East Marshall Street, Richmond, VA 23298 USA; 3 First Clinic of Internal Medicine, Department of Internal Medicine, University of Genoa, viale Benedetto XV 6, 16132 Genoa, Italy; 4 Department of Cardiovascular Sciences, Faculty of Life Sciences and Medicine, King’s College London, London, UK; 5 Cardiovascular Department, Azienda Sanitaria Universitaria Integrata Di Trieste and University of Trieste, Trieste, Italy; 6 BMC Series, Springer Nature, 4 Crinan Street, London, N1 9XW UK

**Keywords:** ESC Congress, ESC guidelines, Heart failure, Hypertension, Myocardial infarction, Pulmonary embolism, Atrial fibrillation, COVID-19

## Abstract

The 2020 annual Congress of the European Society of Cardiology (ESC) was the first ever to be held virtually. Under the spotlight of ‘the cutting edge of cardiology’, exciting and ground-breaking cardiovascular (CV) science was presented both in basic and clinical research. This commentary summarizes essential updates from ESC 2020—The Digital Experience. Despite the challenges that coronavirus disease 2019 (COVID-19) has posed on the conduct of clinical trials, the ESC Congress launched the results of major studies bringing innovation to the field of general cardiology, cardiac surgery, heart failure, interventional cardiology, and atrial fibrillation. In addition to three new ESC guidelines updates, the first ESC Guidelines on Sports Cardiology and Exercise in Patients with Cardiovascular Disease were presented. As former ESC president, Professor Casadei undoubtedly pointed out the ESC Congress 2020 was a great success. During the ESC 2020 Congress, *BMC Cardiovascular Disorders* updated to seven journal sections including Arrhythmias and Electrophysiology, CV Surgery, Coronary Artery Disease, Epidemiology and Digital health, Hypertension and Vascular biology, Primary prevention and CV Risk, and Structural Diseases, Heart Failure, and Congenital Disorders. To conclude, an important take-home message for all CV health care professionals engaged in the COVID-19 pandemic is that we must foresee and be prepared to tackle the dramatic, long-term CV complications of COVID-19 patients.

In 2020, the coronavirus disease 2019 (COVID-19) pandemic dramatically changed the world and way of living. Nevertheless, the 2020 European Society of Cardiology (ESC) annual Congress remained immune, from a scientific point of view, becoming an event like no other, with the most exciting ground-breaking scientific presentations covering the full spectrum of cardiovascular (CV) medicine. The spotlight theme of this virtual ESC Congress was ‘the cutting edge of cardiology’ and it gathered a record-breaking audience of 116,000 delegates from 211 countries [1].

Although daily life has been significantly affected by the pandemic, ‘cardiovascular diseases are still the number one killer worldwide and that as a community we continue to make great strides to improve the lives of our patients’ [1]. The relevance of such a statement is of utmost importance now more than ever, since we failed to foresee the direct and indirect CV consequences of COVID-19 in our patients [2]. Indeed, the fear of COVID-19 strikingly reduced hospital admissions due to both acute coronary syndrome (ACS) and heart failure (HF) up to 50% [3–6]. This in turn, has dramatic consequences for long-term ACS complications and is already associated with increased mortality for both ischemic heart disease and decompensated HF [4, 6]. Therefore, it is important that we prepare for the consequences of the pandemic and thoroughly consider the treatment of CV diseases in the new dimension provided by COVID-19 along while keeping the pace with CV research [7].

Despite being virtual, the Congress attracted a great deal of excellent science, with expected impact on the clinical practice for both cardiologists and non-cardiologists. The ESC Congress shed light on the results of breakthrough studies bringing innovation to the field of cardiology in addition to four new guidelines presented, i.e. those for the management of (1) atrial fibrillation (AF) [8], (2) acute coronary syndromes in patients presenting without persistent ST-segment elevation [9], (3) adult congenital heart diseases [10], and (4) on sports cardiology and exercise in patients with CV disease [11].

In this commentary, we summarized the most important trials presented during the 2020 Virtual ESC Congress (Table 1) which we predict will improve our everyday clinical practice [12–24]. The EMPEROR-Reduced (EMPagliflozin outcomE tRial in Patients With chrOnic heaRt Failure With Reduced Ejection Fraction) trial [23] further strengthened the positive findings observed in the DAPA-HF (Study to Evaluate the Effect of Dapagliflozin on the Incidence of Worsening Heart Failure or Cardiovascular Death in Patients With Chronic Heart Failure) trial [25]. In this study, the anti-diabetic drug empagliflozin on top of optimized medical therapy reduced the composite primary endpoint of CV death and hospitalization in patients with HF and reduced ejection fraction (HFrEF), with the great majority of them presenting with a left ventricle ejection fraction (LVEF) of ≤ 30%. In addition, empagliflozin showed positive effects on reducing the progression of kidney disease [23]. A meta-analysis of EMPEROR-Reduced and DAPA-HF trials confirmed these findings [26].

**Table 1:** Main trials presented during the 2020 Virtual European Society of Cardiology Congress

Trial	References	Study design	General patient characteristics	Number of patients	Main results
EMPEROR-Reduced	Packer et al*.* [23]	Randomized, double-blind, parallel-group, placebo-controlled, event-driven trial	Chronic HF (NYHA class II-IV) with LVEF ≤ 40% (> 70% of patients with LVEF ≤ 30%) receiving appropriate treatment for HF	3730 patients 1863 assigned to empagliflozin 10 mg daily 1867 assigned to placebo	Empagliflozin reduced the primary endpoint (composite of CV death and HHF) by 25% compared with placebo (HR 0.75, 95% CI 0.65–0.86, *p* < 0.001), especially driven by a reduction in HHF (HR 0.75, 95% CI 0.65–0.86, *p* < 0.001) Empagliflozin also reduced the total number of HHF patients (HR 0.70, 95% CI 0.58–0.85, *p* < 0.001) and decreased the decline in eGFR during treatment period (1.73 mL/min/1.73 m^2^/year [95% CI 1.10–2.37, *p* < 0.001]) compared with placebo
PARALLAX	Pieske et al*.* [14]	Randomized, active-controlled, parallel trial	Chronic HF (NYHA II-IV) with LVEF > 40% receiving appropriate treatment for HF and elevated NT-proBNP at screening	2572 patients 1286 assigned to sacubitril/valsartan (1281 for full analysis) 1286 assigned to IMT (1285 for full analysis)	Sacubitril/valsartan reduced one of the first primary co-outcome—NT-proBNP change from baseline to week 12—compared with IMT (AGMR 0.84, 95% CI 0.80–0.88) *p* < 0.0001). However, the other primary co-outcome—6MWT change from baseline to week 24—was not met when comparing sacubitril/valsartan to IMT (AMD − 2.5, 95% CI − 8.53 to 3.53, *p* = 0.24) Among secondary outcomes, sacubitril/valsartan slowed eGFR decrease compared with IMT (− 1.47 ml/min/1.73 m^2^ vs. − 2.57 ml/min/1.73 m^2^, *p* = 0.016) and reduced death due to cardiac failure or HHF (HR 0.64, 95% CI 0.42–0.97, *p* = 0.034)
HOME-PE	Roy et al*.* [15]	Randomized, controlled, parallel, open-label trial	Patients with PE followed for 90 days	1970 patients 984 assigned to HESTIA group 986 assigned to sPESI group	The HESTIA rule was non-inferior to the sPESI score with respect to the primary composite outcome of all-cause death, recurrent VTE, or major bleeding at 30 days (3.8% vs. 3.6%, *p* for non-inferiority = 0.005) The two strategies showed no differences in the proportion of patients treated at home or early discharged (38.4% for HESTIA group vs. 36.6% for PESI group, p for superiority = 0.41) HESTIA rule identified less patients as eligible for outpatient management compared with sPESI score (39.4% vs. 48.4%), but it did better for those patients treated as outpatients among eligible patients (88.4% vs. 64.8%)
COLCOT	Bouabdallaoui et al*.* [17]	Randomized, double-blind trial	Patients with MI within the last 30 days	4745 patients 2366 assigned to colchicine 0.5 mg once daily 2379 assigned to placebo	Among patients who suffered a recent MI, low-dose colchicine was effective at reducing MACEs (CV death, MI, stroke, resuscitated cardiac arrest, or urgent hospitalization for UA leading to revascularization) which occurred in 5.5% of the colchicine group compared with 7.1% of the placebo group (HR 0.77, 95% CI 0.61–0.96, *p* = 0.02). Nevertheless, higher non-CV mortality with colchicine was noted
EVAPORATE	Budoff et al*.* [19]	Randomized, double-blind, placebo-controlled trial	Patients with angiographic CAD on statins	80 patients 40 assigned to icosapent ethyl 4 g/day 40 assigned to placebo	Icosapent ethyl reduced the primary endpoint—LAP volume at 18 months—compared with placebo (− 0.3 vs. 0.9 mm^3^, *p* = 0.006)
LoDoCo 2	Nidorf et al*.* [21]	Randomized, placebo-controlled trial	Patients with CHD	5522 patients 2761 assigned to colchicine 0.5 once daily 2761 assigned to placebo	Colchicine improved CV outcomes among patients with CCD compared with placebo during 1 month follow-up. Colchicine prevented CV death, MI, stroke, ischemia-driven revascularization when compared to placebo (6.8% vs. 9.6%; HR 0.69, 95% CI 0.57–0.83, *p* < 0.001).Nevertheless, higher non-CV mortality with colchicine was noted
COPS	Tong et al*.* [24]	Randomized, double-blind, placebo-controlled trial	Patients with ACS on statins	795 patients 396 assigned to colchicine 0.5 twice daily for 1 month, then 0.5 mg once daily for 11 months 399 assigned to placebo	Colchicine did not improve CV outcomes (death from any cause, ACS, ischemia-driven urgent revascularization and non-cardioembolic ischemic stroke) compared with placebo in patients with ACS after 1 year (6.1% vs. 9.5%, *p* = 0.09). Nevertheless, higher non-CV mortality with colchicine was noted
BRACE-CORONA	Renato et al*.* [13]	Randomized, parallel trial	Patients with COVID-19 and ACE-is/ARBs	659 patients 334 stopping ACE-is/ARBS 325 continuing ACE-is/ARBs	Suspending ACE-Is/ARBs compared with continuing them did not improve the days alive and out of the hospital (22.9 days in the continuing ACEI/ARB group vs. 21.9 days in the suspending ACEI/ARB group, *p* = 0.09)
EXPLORER-HCM	Olivotto et al*.* [22]	Randomized, double-blind, placebo controlled, parallel trial	Patients with HCM with LVOT gradient ≥ 50 mmHg and NYHA class II-III symptoms	251 patients 123 assigned to mavacamten (starting dose 5 mg) for 30 weeks 128 assigned to placebo for 30 weeks	The primary endpoint (≥ 1.5 mL/kg/min increase in pVO_2_ and at least 1 NYHA class reduction or a ≥ 3.0 mL/kg/min pVO_2_ increase without NYHA class worsening) was met by 37% of patients on mavacamten versus 17% of those on placebo (difference + 19.4%, 95% CI 8.7–30.1, *p* = 0.0005) Secondary endpoints were also met, with patients on mavacamten experiencing reduction in post-exercise LVOT gradient, increase in pVO_2_, and improvement in in symptom scores compared with placebo (*p* < 0.001 for all) Safety and tolerability were comparable between groups
POPular TAVI	Brouwer et al*.* [18]	Randomized, open-label, controlled trial	Patients undergoing TAVI without indication for long-term AC	665 patients 331 assigned to aspirin alone for 3 months 334 assigned to aspirin and clopidogrel for 3 months	One co-primary outcome—all bleedings—occurred in 15.1% of patients on aspirin versus 26.6% of those on aspirin and clopidogrel (RR 0.57, 95% CI 0.42–0.77, *p* = 0.001). The second co-primary outcome—non-procedure-related bleeding across 12 months—was observed in 15.1% of patients on aspirin versus 24.9% of those on aspirin and clopidogrel (RR 0.61, 95% CI 0.44–0.83, *p* = 0.005) Composite secondary endpoints (death from CV causes, non-procedure-related bleeding, stroke, or MI; death from CV causes, ischemic stroke, or MI) occurred less frequently in patients assigned to aspirin alone compared with those assigned to aspirin and clopidogrel (*p* < 0.05 for both)
REALITY	Steg et al*.* [16]	Randomized, parallel trial	Patients with acute MI and Hgb ≤ 8 to ≤ 10 g/dL at admission	666 patients 342 assigned to liberal (Hgb ≤ 10 g/dL, goal Hgb > 11 g/dL) RBC transfusion 324 assigned to restrictive (Hgb ≤ 8 g/dL, goal Hgb 8–10 g/dL) RBC transfusion	Primary outcome—all-cause death, reinfarction, stroke, and emergency revascularization prompted by ischemia, was 11.0% versus 14.0% for restrictive versus liberal transfusion strategy (HR 0.77, 95% CI 0.50–1.18, p < 0.05 for non-inferiority, *p* = 0.22 for superiority) Among secondary outcomes, infections and ALI were higher in those assigned to a more liberal strategy
EAST-AFNET 4	Kirchhof et al*.* [20]	Randomized, parallel trial	Patients with new-onset or untreated, early AF (diagnosis within 1 year) and concomitant CV conditions	2789 patients 1395 assigned to rhythm therapy 1394 assigned to usual care	Rhythm control reduced the primary outcome—CV death, stroke, HHF, or ACS, for rhythm control versus usual care—compared with usual care (HR 0.79, 95% CI 0.66–0.94, *p* = 0.005). The trial was stopped early due to efficacy
RATE-AF	Kotecha et al*.* [12]	Randomized, open label, parallel trial	Patients with permanent AF	160 patients 80 assigned to digoxin 80 assigned to β-blocker (bisoprolol)	No difference was observed between digoxin and β-blocker for the primary outcome -patient-reported quality of life at 6 months Digoxin improved some quality of life measures at 12 months and was associated with greater reductions in NYHA class and NT-proBNP levels Fewer adverse events were observed for those treated with digoxin compared with β-blocker (29 vs. 142 events, *p* < 0.001)
CASA-AF	Haldar et al*.* [29]	Randomized, parallel, controlled trial	Patients with long-standing persistent AF	120 patients 80 assigned to surgical ablation 60 assigned to catheter ablation	No group differences on primary outcomes observed at 12 months—freedom from AF, AF burden or serious adverse effects. One death in surgical group. Improved symptomatic relief, cost-effectiveness, and quality of life in catheter group

With regard to inflammation and CV disease, the LoDoCo2 (Low Dose Colchicine for secondary prevention of cardiovascular disease) trial showed that in patients with chronic coronary disease, low-dose colchicine (0.5 mg once daily) significantly reduced the risk of CV events compared with placebo [21]. These results confirmed the previous observations from the open-label LoDoCo (Low Dose Colchicine) trial, that found a reduced risk of acute CV events among those patients with chronic coronary disease who received 0.5 mg of colchicine once daily than the placebo group [27]. These beneficial effects were first observed in the COLCOT (Colchicine Cardiovascular Outcomes Trial), where patients with a recent acute myocardial infarction (within 30 days) who received colchicine 0.5 mg once daily experienced less frequently the composite endpoint of CV death, resuscitated cardiac arrest, myocardial infarction, stroke, or urgent hospitalization for angina leading to coronary revascularization compared with those who received placebo [28].

Interesting news came from the AF field, not only due to the publication of the updated guidelines [8], but also from the results of three major studies—the EAST-AFNET 4 (Early Treatment of Atrial Fibrillation for Stroke Prevention Trial), RATE-AF (Rate Control Therapy Evaluation in Permanent Atrial Fibrillation), and CASA-AF (Catheter Ablation Versus Thoracoscopic Surgical Ablation in Long Standing Persistent Atrial Fibrillation) [29]. The EAST-AFNET 4 found that the early initiation of a rhythm control therapy was associated with a reduced risk of CV outcomes (death from CV causes, stroke, hospitalization for HF, or ACS) than usual care in those patients with early AF and other CV diseases across a 5-year follow-up period [20]. The RATE-AF trial gave a new life to an old, sometimes neglected, drug like digoxin. Indeed, this study showed that digoxin was associated with symptom improvement and reduction in N-terminal-pro B-type natriuretic peptide compared with a β-blocker in patients with permanent AF [12]. CASA-AF demonstrated that surgical ablation was not superior to catheter ablation in treating long-standing persistent AF, while catheter ablation allowed greater symptomatic relief, cost-effectiveness, and quality of life [29].

A relevant trial in these challenging times of COVID-19 was presented at the ESC Congress, the BRACE CORONA (Angiotensin Receptor Blockers and Angiotensin-converting Enzyme Inhibitors and Adverse Outcomes in Patients With COVID19) trial. This study tested the temporary suspension of renin–angiotensin–aldosterone system (RAAS) blockers for 30 days versus continuation of these drugs in those chronically taking RAAS blockers and hospitalized due to COVID-19 [13]. The conclusion of the study was that among patients hospitalized with COVID-19 and receiving chronic a RAAS blocker, drug suspension was not beneficial and suspension versus continuation did not improve the days alive and out of the hospital [13].

Lastly, a game changing new molecule, mavacamten, a selective allosteric inhibitor of cardiac myosin ATPase, improved exercise capacity, left ventricular outflow tract obstruction, New York Heart Association (NYHA) functional class, and health status in patients with obstructive hypertrophic cardiomyopathy. The results of the EXPLORER-HCM trial might reshape, in the future, the landscape of hypertrophic cardiomyopathy, significantly reducing the number of patients with the obstructive form of the disease and, perhaps, ameliorating long-term outcomes and reducing the risk of sudden death in these patients [22].

During the 2020 Virtual ESC Congress, the Editorial Board of *BMC Cardiovascular Disorders* met. This virtual meeting was led by the Editor, Dr. Ciarán Martin Fitzpatrick, together with Sections Editors and most Associate Editors. The meeting provided the opportunity to discuss the positive changes at the Journal and the future steps to keep progressing. The improvement in the handling of manuscripts by Associate Editors greatly reduced turnaround times and attracted more submissions in the past months. As far as the metrics is concerned, *BMC Cardiovascular Disorders* steadily improved from previous years with an updated 2019 yearly downloads of 890,588 and impact factor of 2.078. The Journal has also updated its sections to comply with new relevant topics in CV medicine. The new sections are: Arrhythmias and Electrophysiology; CV Surgery; Coronary Artery Disease; Epidemiology and Digital Health; Hypertension and Vascular Biology; Primary Prevention and CV Risk, and Structural Diseases, Heart Failure, and Congenital Disorders. Presented during this meeting was Lesyuk et al*.*’s ‘Cost-of-illness studies in heart failure: a systematic review 2004–2016’ [30], which was the journal’s most cited article so far in 2020 with 37 citations.

Together with this exciting news, *BMC Cardiovascular* is pleased to offer its readers a wide range of submission initiatives, which are helpful in the progression of knowledge within the CV field. We must bear in mind that despite the world locking down momentarily, the continuous effort of healthcare professionals and researchers contributed to timely treatments and a better understanding of the COVID-19 disease and its direct and indirect impact on patients’ management, treatment, outcomes and, ultimately, our daily life.

## Data Availability

Not applicable.

## Abbreviations

AC: Anticoagulation
ACE-i: Angiotensin-converting enzyme inhibitor
ACS: Acute coronary syndrome.
AF: Atrial fibrillation
AGMR: Adjusted geometric mean ratio
ALI: Acute lung injury
AMD: Adjusted mean difference
ARB: Angiotensin-receptor blocker
CAD: Coronary artery disease
COVID-19: Coronavirus disease 2019
CCD: Chronic coronary disease
CHD: Coronary artery disease
CI: Confidence interval
CV: Cardiovascular
eGFR: Estimated glomerular filtration rate
HF: Heart failure
Hgb: Hemoglobin
HHF: Hospitalization for heart failure
HR: Hazard ratio
IMT: Individualized medical therapy
LAP: Low-attenuation plaque
LVEF: Left ventricle ejection fraction
MACE: Major adverse cardiac event
MI: Myocardial infarction
NYHA: New York Heart Association
pVO_2_: Peak oxygen consumption
RBC: Red blood cell
RR: Risk ratio
sPESI: Simplified Pulmonary Embolism Severity Index
TAVI: Transcatheter aortic-valve implantation
UA: Unstable angina
